# Genetic profiling for diffuse type and genomically stable subtypes in gastric cancer

**DOI:** 10.1016/j.csbj.2020.10.021

**Published:** 2020-10-29

**Authors:** Yiwei Ling, Yu Watanabe, Mayuki Nagahashi, Yoshifumi Shimada, Hiroshi Ichikawa, Toshifumi Wakai, Shujiro Okuda

**Affiliations:** a Division of Bioinformatics, Niigata University Graduate School of Medical and Dental Sciences, 1-757 Asahimachi-dori, Chuo-ku, Niigata 951-8510, Japan; b Division of Digestive and General Surgery, Niigata University Graduate School of Medical and Dental Sciences, 1-757 Asahimachi-dori, Chuo-ku, Niigata 951-8510, Japan

**Keywords:** Gastric cancer, Genomically stable, Diffuse type, Mutational signature, Lauren classification

## Abstract

Gastric cancer is one of the most common and clinically important diseases worldwide. The traditional Laeuren classification divides gastric cancer into two histopathological subtypes: diffuse and intestinal. Recent cancer genomics research has led to the development of a new classification based on molecular characteristics. The newly defined genomically stable (GS) subtype shares many cases with the histopathologically diffuse type. In this study, we performed genetic profiling of recurrently and significantly mutated genes in diffuse type and GS subtype tumors. We observed significantly different genetic characteristics, although the two subtypes overlapped in many cases. In addition, based on the profiles of the significantly mutated genes, we identified molecular functions and mutational signatures characteristic of each subtype. These results will advance the clinical application of the diffuse type and GS subtype gastric cancer in precision medicine for treating gastric cancer.

## Graphical abstract

**Figure:**
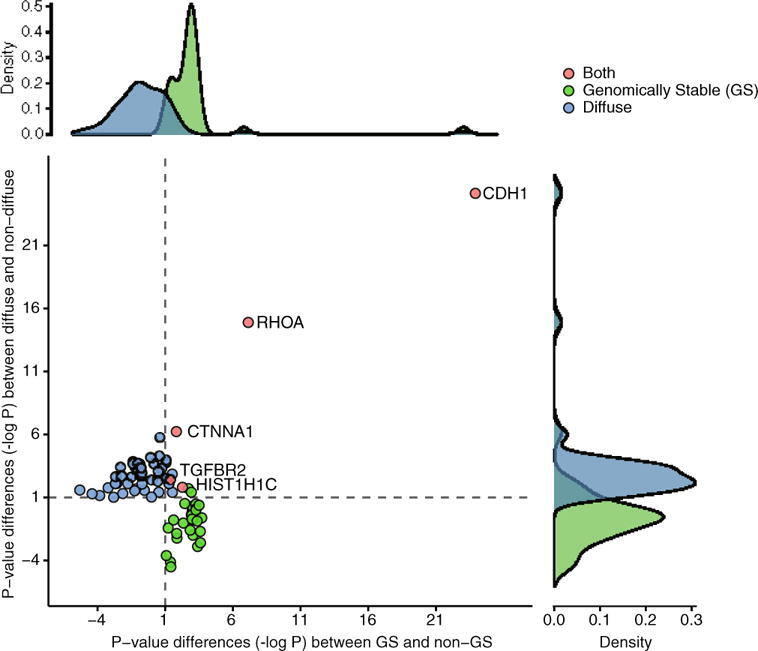

## 1 Introduction

Gastric cancer (GC) is the third leading cause of death worldwide, with hundreds of thousands of people lost each year [1], [2]. The incidence of GC is highest in East Asia and lowest in North America [2], [3]. GC is a heterogeneous disease with phenotypic diversity that is traditionally divided into two groups, intestinal and diffuse types, according to the Lauren classification [4]. This traditional classification has not shown clear clinical utility to date. The poorly differentiated diffuse type is infiltrated with abundant stroma, progresses faster than the intestinal type, and is associated with a poor prognosis [5], [6]. Recently, next- generation sequencing technology has revealed an extensive repertoire of potentially cancer driver genes; thus the landscape of mutations in GC has also been disclosed [7], [8], [9], [10]. The Cancer Genome Atlas (TCGA) project classified GC into four molecular subtypes; Epstein-Barr virus-positive (EBV), microsatellite instability (MSI), genomically stable (GS), and chromosomal instability (CIN) subtypes [7], [11]. These recent studies focused on molecular classification by hypothesizing that tumor classification based on molecular data is more clinically influential than traditional histopathological classification in terms of selecting treatment methods and predicting patient prognosis. This molecular classification has improved our understanding of the molecular profile and heterogeneity in GC. However, these classifications are not designed to optimize patient selection for targeted therapies and their clinical utilities are also unknown in many cases.

The GS subtype is difficult to clearly characterize based on the pattern of gene mutations because the mutations are sporadic and present in low numbers. However, many cases of GS are classified as the diffuse type according to the Lauren classification, and most of cases are shared between the diffuse type and GS subtype [7], [11]. The TCGA gastric cancer study also showed that 73% of diffuse type cases can be classified as the GS subtype, suggesting that the genetic features of GS are associated with the diffuse phenotype [7]. Mutations in genes such as *CDH1* and *RHOA* have been found to be particularly prominent in the diffuse type, and their relevance to the mechanism of carcinogenesis has been studied [12], [13], [14]. However, it is difficult to discuss the diffuse type in terms of other specific gene mutations, as many cases overlap with the GS subtype and show few characteristic gene mutations.

Here, we statistically investigated the characteristics of gene mutations in these two subtypes of GC, diffuse and GS. We identified significantly mutated and possible driver genes in each type. We observed five significantly mutated genes common to both subtypes and significantly mutated genes specific to each subtype. Despite overlap in the characteristics of the subtypes, the characteristics of the pattern of gene mutations were quite different in each subtype. In addition, each subtype was characterized based on functional features and mutation signature analysis associated with the gene mutation patterns. The patterns and characteristics of the gene mutations identified in this study are useful for developing clinical treatments for each type of GC.

## 2 Materials & methods

### 2.1 Mutation data

Gene mutation data of TCGA-STAD stomach adenocarcinoma were downloaded from the cBioPortal for Cancer Genomics (https://www.cbioportal.org). Of these mutation data, cases with classification results identified in TCGA GC study [7] were extracted (n = 295). According to the clinical information download from cBioPortal, these cases were classified into the Epstein-Bar virus-positive (n = 26), MSI (n = 64), GS (n = 58), and chromosomal instability (n = 147) subtypes by TCGA classification as well as diffuse (n = 69), intestinal (n = 196), mixed (n = 19) and unknown (n = 11) types according to the Lauren classification. Of these, samples with somatic mutation data were extracted (n = 289). All obtained cases were divided into diffuse (n = 67) and non-diffuse (n = 192) subtypes and GS (n = 55) and non-GS (n = 234) subtypes. Cases with mixed and unknown types were classified according to the Lauren classification. Cases labeled as “non-diffuse” did not include the mixed and unknown types in subsequent analysis.

### 2.2 Significantly mutated genes

We identified mutated genes as those with non-synonymous mutation frequencies greater than that of the background. For this calculation, we estimated the expected number of protein-altering mutations in each gene, which is affected by the gene length and background mutation rate. First, the number of non-synonymous mutation sites (nonsynonymous SNV or short Indel) was counted for each gene. Second, the background mutation rate was calculated as the frequency by dividing the total number of observed mutations in all genes by the total gene length, which was then used to determine whether the observed mutation count in a gene was higher than the expected number. The expected number of mutations in each gene was estimated as the total number of observed mutations in all genes and the background mutation rate. Finally, genes with more observed mutations than the expected number were statistically tested by right-tailed Poisson tests [15], [16], which were conducted using rateratio.test in R package (https://www.r-project.org). P-values were adjusted for multiple testing using the Storey method [17] with R. Significantly mutated genes showing 10-fold P-value differences between the diffuse and non-diffuse type, and the GS and non-GS subtypes were extracted.

### 2.3 Molecular interaction network analysis

The molecular interaction and association network data defined in the STRING database [18] were used. The interaction network data of genes with 10-fold P-value differences for the diffuse-type and GS subtype were obtained. The edge number between the diffuse type and GS subtype was 81, whereas the specific edge numbers in these two types were 93 and 69, respectively. Statistical test (paired *t*-test) for the fraction of in-edges linked within each type and inter-edges linked these two different types was calculated. The diffuse and GS genes with 10-fold P-value differences had significantly greater frequencies of in-edges than inter-edges (P-value: 2.48 × 10^−5^ and 0.0016, respectively). Finally, interaction networks were constructed using Gephi graph network visualizing software [19].

### 2.4 Gene set enrichment analysis

Significantly mutated genes with the top 50P-value differences were subjected to g:Profiler analysis [20]. g:Profiler analysis was performed to obtain detailed information on biological functions and pathways that significantly enriched significantly mutated genes with differences in P-values in the diffuse type and GS subtype. g:Profiler analysis can provide several types of results based on representative databases such as Gene Ontology [21], KEGG [22], Reactome [23], and WikiPathway [24].

### 2.5 Mutational signature analysis

Mutational signatures of the diffuse and non-diffuse types and GS and non-GS subtypes were analyzed as follows: each single-nucleotide variant was classified in a matrix of the 96 possible substitutions based on the sequence context comprising the nucleotides 5′ and 3′ to the position of the mutation. Mutational signatures were extracted by non-negative matrix factorization analysis with the SomaticSignatures R package [25] and plotted with the ggplots R package (http://ggplot2.org/). The mutational signature classifications defined by the COSMIC database [26] were used in this analysis.

## 3 Results

### 3.1 Overview of GC used in this study

**Fig. 1:**
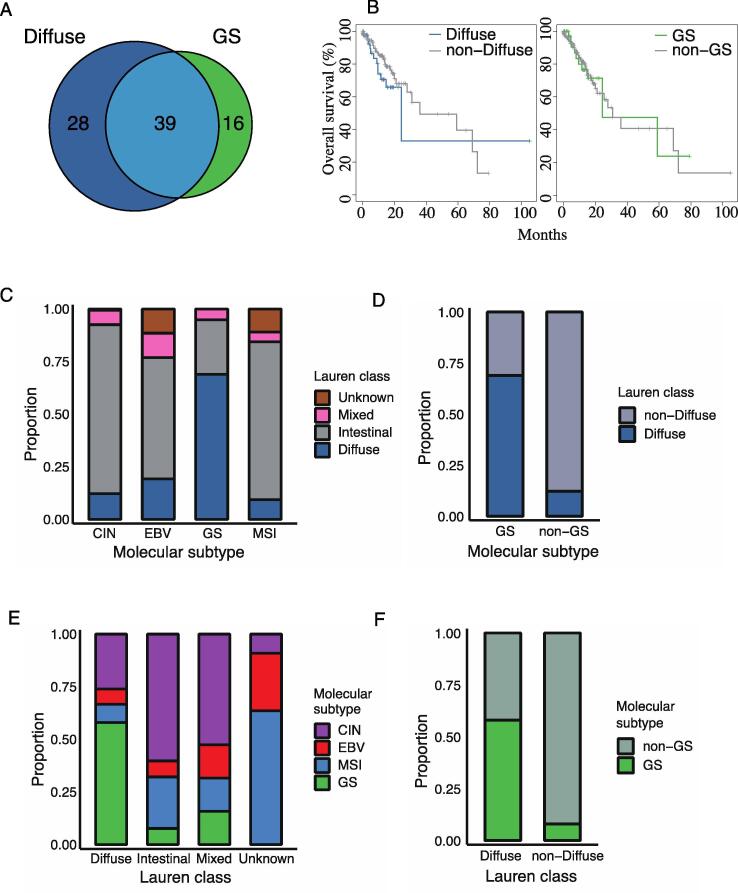
Overview of the diffuse type and GS subtype used in this study. (A) The number of sequenced cases in the diffuse type and GS subtype. (B) Survival plots for the diffuse type and GS subtype. Overall survival time was obtained from TCGA. (C–F) Distribution of cases in the diffuse type and GS subtype based on the Lauren classification and TCGA molecular classification.

In the TCGA GC data, 67 and 55 cases were the diffuse type and GS subtype, respectively, among the 289 total cases (Fig. 1A). Thirty-nine cases were defined as having both diffuse type and GS subtype characteristics, accounting for 58% of diffuse type and 71% of GS subtype. Survival plots for these subtypes showed a slightly worse trend in prognosis for the diffuse versus non-diffuse type (P = 0.30), whereas there was no difference for GS compared to the non-GS subtype (P = 0.90) (Fig. 1B). Among the four molecular subtypes defined in the TCGA GC study, the distribution of the diffuse type was the most prevalent for the GS subtype, as described above, and 10–20% of the other three molecular subtypes were classified as the diffuse type (Fig. 1C and D). In construct, 57% of cases classified as the diffuse type according to the Lauren classification were of the GS subtype and only a few percent of the GS subtype cases were found among the non-diffuse type (Fig. 1E and F). Detailed percentages in Fig. 1C-F are summarized in Supplementary Table 1.

### 3.2 Significantly mutated genes in GC

**Fig. 2:**
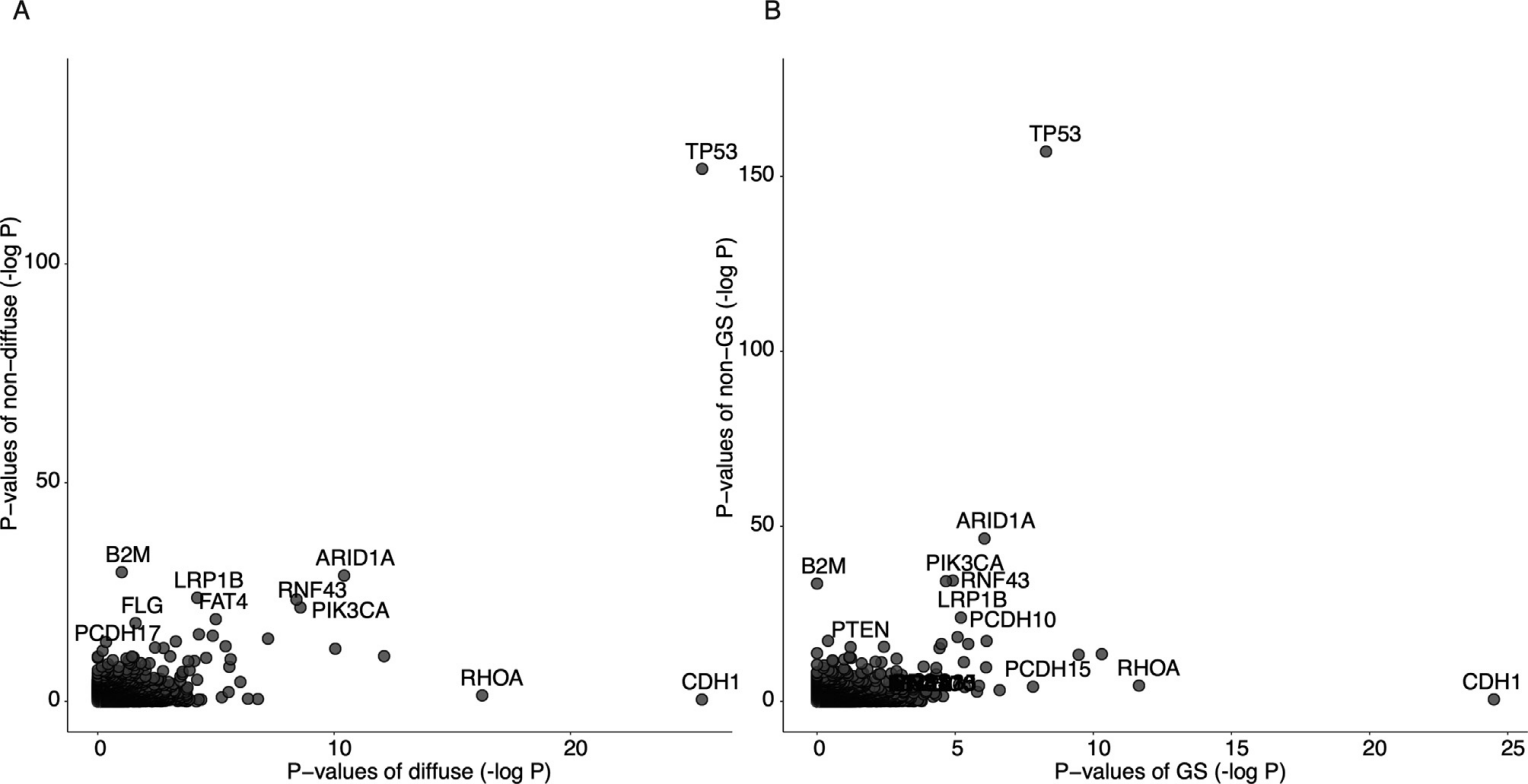
Distribution of P-values of significantly mutated gene detection. (A) P-values in the diffuse against non-diffuse type and (B) P-values in the GS against non-GS subtype. All the P-values were transformed to negative log10 P-value.

We statistically evaluated whether the frequency of mutations in a specific gene was significantly more higher than in other background genes. We identified genes with significant mutation frequencies in the diffuse and non-diffuse types and the GS and non-GS subtypes (Supplementary Table 2). Fig. 2A and B show the distribution of negative log P-values between diffuse vs. non-diffuse and GS vs. non-GS, respectively. We identified 81 genes in the diffuse type (P < 0.01, Q < 0.25), and 68 genes in the GS subtype (P < 0.01, Q < 0.25) as significantly mutated. In both subtypes, *CDH1* and *RHOA* showed extremely low P-values compared with the non-diffuse type and non-GS subtype, whereas *TP53* showed significant P-values in all four categories, including non-diffuse and non-GS. Thus, *TP53* is thought to be associated with GC overall rather than with the diffuse type and GS subtype.

**Fig. 3:**
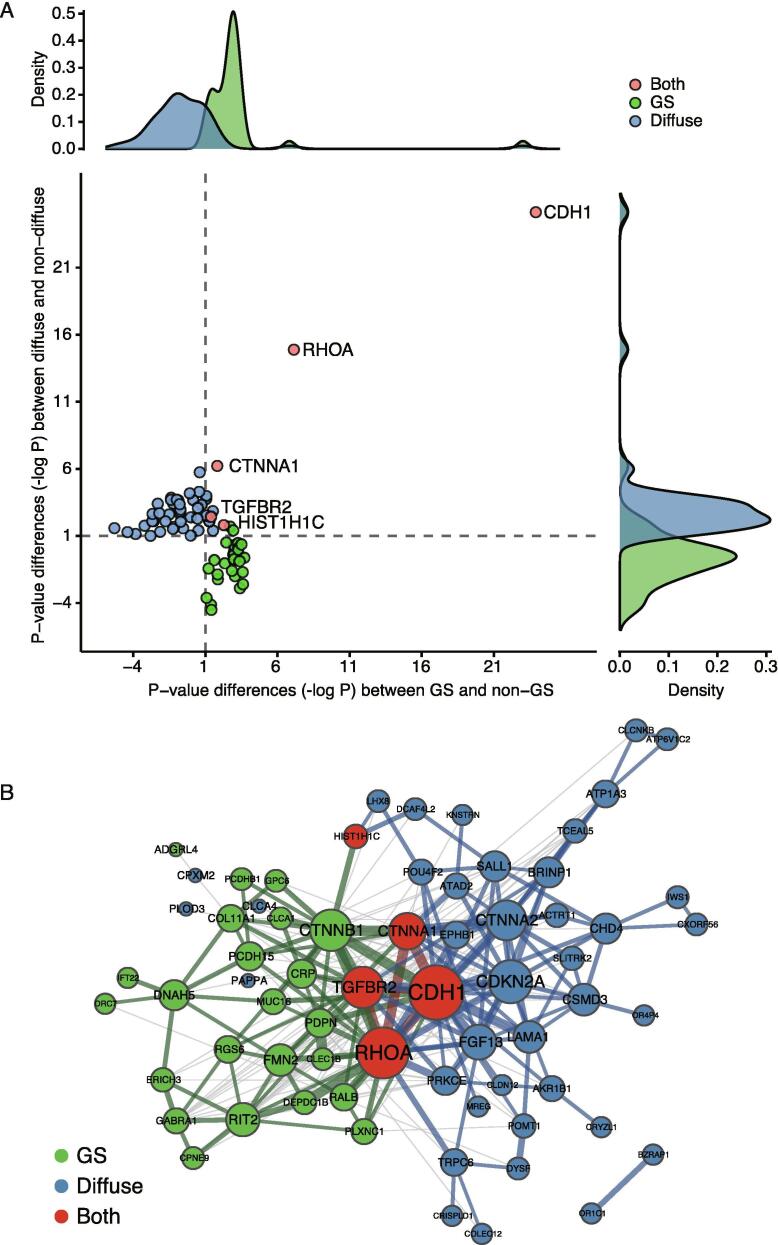
Distribution of differences between P-values in the diffuse type and GS subtype. (A) Scatter plot of P-value differences in the diffuse type and GS subtype. All P-values were transformed to negative log10 P-value. (B) Molecular interaction network of genes with P-value differences in the diffuse type and GS subtype. Circle size represents the number of interactions for each gene and width of an edge indicates the score as defined in the STRING database. Genes showing 10-fold P-value differences in the diffuse type and GS subtype are shown in green and blue, respectively. Red circles indicate the common significantly mutated genes in both subtypes. Blue and green edges indicate an in-edge linked in the diffuse type and GS subtype, and a gray edge indicates an inter-edge between these types. (For interpretation of the references to colour in this figure legend, the reader is referred to the web version of this article.)

Next, we extracted genes with differential P-values, for which the frequency of mutations was sufficiently large for the diffuse type and GS subtype compared to their respective non-diffuse and non-GS counterparts. Genes showing P-values with a difference of at least 10-fold were extracted. Of these significantly mutated genes in the diffuse type and GS subtype, those with greater P-values in the counterpart subtype were extracted and the negative logarithm of the difference in P-values was calculated. As a result, 51 and 35 genes remained in the diffuse type and GS subtype, respectively (Supplementary Fig. 1). Five genes, *CDH1*, *RHOA*, *HIST1H1C*, *TGFBR2* and *CTNNA1*, were significantly mutated in both subtypes (Fig. 3A). Furthermore, when genes showing a 100-fold difference were extracted, only *CDH1* and *RHOA* remained as common genes. Two genes, *CDH1* and *RHOA*, exhibited extremely low P-values in the diffuse type and GS subtype compared to their counterparts and thus may be strongly associated with the two subtypes. *CDH1* showed P-values of 2.74e-26 and 0.3696 in diffuse and non-diffuse types, respectively, and 3.17e-25 and 0.2588 in GS and non-GS subtypes, respectively. *RHOA* also exhibited extremely low P-values of 5.42e-17 and 2.22e-12 in the diffuse type and GS subtype, respectively; these values were much lower than those of 0.0416 and 3.03e-05 observed in their non-diffuse and non-GS counterparts. The mutated genes associated with these subtypes and distribution of each case are shown in Supplemental Fig. 2 as an oncoprint. In addition, molecular interaction and association network analysis was performed for genes showing P-value differences. The results showed that the diffuse type and GS subtype were clearly distinguished (Fig. 3B). A diffuse and GS-specific network was connected for the common genes, particularly for the *CDH1*, *RHOA*, *TGFBR2*, and *CTNNA1* genes.

### 3.3 Enrichment analysis of significantly mutated genes in diffuse-type and GS subtypes

**Fig. 4:**
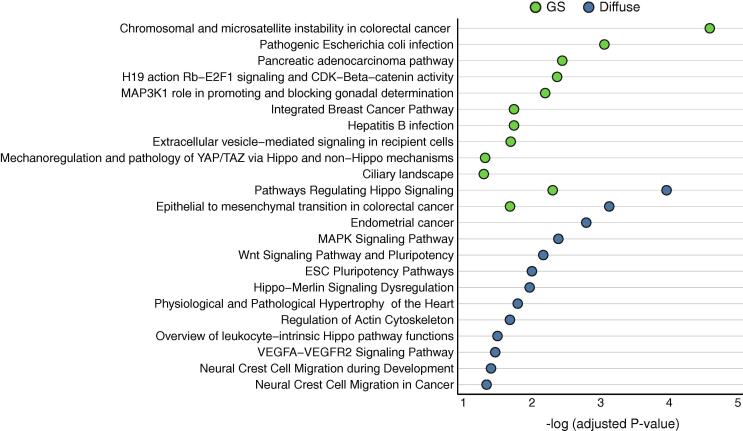
Enriched functions/pathways of significantly mutated genes in the diffuse type and GS subtype. Enrichment analysis was performed using the g:Profiler tool and the categories defined by WikiPathway were used.

Enrichment analysis of the molecular functional distribution was performed for genes showing differences in P-values of 10-fold (Supplementary Table 3). WikiPathway clearly distinguished the molecular functions/pathways of the diffuse type and GS subtype (Fig. 4). The results showed that “pathways regulating hippo signaling,”, “epithelial to mesenchymal transition in colorectal cancer”, “MAPK signaling pathway”, “Wnt signaling pathway and pluripotency”, “ESC pluripotency pathways”, “Hippo–Merlin signaling dysregulation”, “neural crest cell migration during development”, “neural crest cell migration in cancer” and others were significantly enriched in the diffuse type, whereas “chromosomal and microsatellite instability in colorectal cancer”, “pathogenic *Escherichia coli* infection”, “H19 action Rb–E2F1 signaling and CDK–beta–catenin activity”, “hepatitis B infection”, “ciliary landscape” and others were enriched in the GS subtype. Thus, the diffuse type was likely to have mutations in functional pathways related to cell migration and pluripotency. In contrast, more frequent mutated genes in the GS subtype functions in cell cycle-related signaling pathways and bacterial infection. For comparison with the results of the top P-value differences described above, the top 100 genes with significant P-values in GS and diffuse types examined by enrichment analysis. The enriched functional categories between the GS subtype and diffuse type were quite similar (Supplementary Table 4).

### 3.4 Mutational signature analysis to explore causal factors

**Fig. 5:**
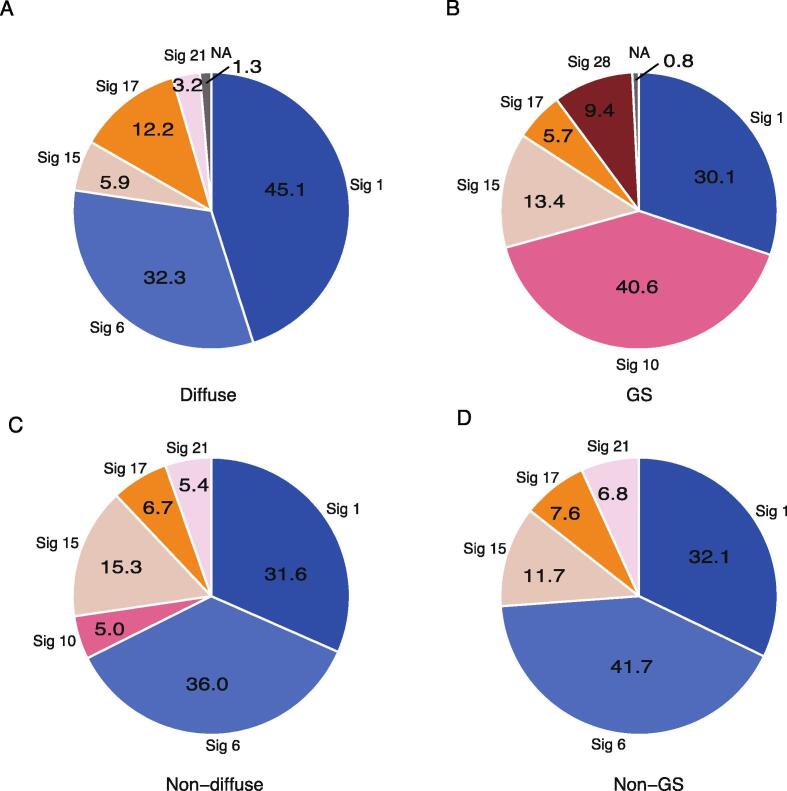
Mutational signatures of diffuse type and GS subtype. (A) Mutational signature distribution calculated from mutations found in the diffuse type, (B) GS subtype, (C) non-diffuse type, and (D) non-GS subtype.

We performed mutational signature analysis to identify the cause of gene mutation (Fig. 5, Supplementary Fig. 3). This method is used to investigate the molecular mechanisms of carcinogens, such as ultraviolet light and smoking, and molecular functions such as homologous recombination and mismatch-repair types, by using the patterns of three bases including DNA substitution and the flanking bases. Mutational signature analysis identified signatures 1, 6, 15, and 17 as common to all the diffuse type and GS subtype, and as well as their counterparts. In contrast, only signature 21 was identified outside of the GS subtype, as a feature could not be observed in the GS subtype. Signature 28 was identified only in the GS subtype and was distinguished from the other subtypes in this regard. Signature 10 was detected as a signature related to hypermutation in the GS subtype and non-diffuse types.

## 4 Discussion

The subtypes of diffuse and GS are distinguished based on histopathological classification and molecular classification according to gene mutations, although most cases share various features (Supplementary Table 4). However, the results of statistical analysis of the frequency of gene mutations were specific to each type, and divided into groups of differentially mutated genes, except for five common genes. Furthermore, using stringent criteria (100-fold difference in P-values relative to the counterparts), two highly mutated genes, *CDH1* and *RHOA*, were detected in both subtypes, which is supported by many previous genomic studies of GC [12], [13], [14]. Significant and highly mutated genes, which are common among these subtypes, may characterize each subtype of cancer. Thus, based on clinical characteristics, the diffuse type is poorly differentiated, and the genes involved in poor differentiation and increased malignancy are more frequently mutated and associated with molecular functions and pathways that promote cancer metastasis. In contrast, the GS subtype is associated with mutations in genes involved in cell proliferation, which may cause differentiated cancers. Consistent with this hypothesis, enrichment analysis indicated that the diffuse type is strongly associated with functions such as pluripotency and cell migration, which are suggested to be associated with a poorly differentiated cancer. In addition, the GS subtype was shown to be enriched in the functions of bacterial infection and cell cycle, suggesting an association with cell proliferation as a differentiated form of cancer. This observation is also consistent with the above hypothesis. Therefore, the results of enrichment analyses of molecular functions and pathways may explain these diffuse and GS types of cancer.

The results of the mutational signature analysis revealed different signatures for the diffuse type and GS subtype. Signature 10, which showed *POLE* mutation type hypermutation, was more prominent in the GS subtype mutational signatures. This is because one donor with the GS subtype had a *POLE* mutation. Although the donor with the *POLE* mutation exhibited a hypermutation type [26] and was classified as the hypermutation (MSI status is high) subtype in the four molecular classification systems defined in TCGA GC study [7], this patient was not classified as a hypermutation but rather as GS because of the stable MSI status. This patient showed a large number of mutations (>200 tumor mutation burden) and may be classified as the hypermutation subtype. However, in this study, the original classification result defined in the TCGA GC study was used, and signature 10 was observed in the GS subtype.

Furthermore, signature 21 in the diffuse type and signature 28 in GS were remarkably different between the diffuse type and GS subtype. Signature 21 has also been observed in the non-diffuse type and non-GS subtypes, whereas signature 28 has been observed only in the GS subtype and can be used to characterize this subtype. Signatures 21 and 28 are defined by the COSMIC database as commonly found in GC, but the associated etiology is unknown [26]. Although the causal factors are unknown, the two different signatures 21 and 28 are likely extremely important factors in the etiology for distinguishing the histopathology between the two subtypes.

The diffuse type was characterized by genes that may also be involved in molecular function and prognosis, whereas the GS subtype is characterized by a mutational signature that indicates the cause of the cancer. Our findings and the information on the pattern of genetic mutations obtained in this study are useful for future clinical studies of treatments for these two subtypes of diseases.

## 5 Conclusions

Although the diffuse type of poor prognosis and GS subtype of sporadic gene mutations have many histopathologically and genetically common features and cases in GC, we found a clear difference in mutation profiles between these two GC subtypes. In addition, we observed clear differences in their associated molecular functions and possible causal mutational signatures. Clinical applications based on these statistically supported genetic mutation profiles are needed to apply these results in medicine to treat GC.

## CRediT authorship contribution statement

**Yiwei Ling:** Investigation, Methodology, Software, Funding acquisition, Writing - original draft. **Yu Watanabe:** Software, Visualization. **Mayuki Nagahashi:** Data curation, Funding acquisition. **Yoshifumi Shimada:** Data curation. **Hiroshi Ichikawa:** Data curation. **Toshifumi Wakai:** Data curation, Conceptualization. **Shujiro Okuda:** Supervision, Writing - review & editing, Funding acquisition, Project administration.

## Declaration of Competing Interest

The authors declare that they have no known competing financial interests or personal relationships that could have appeared to influence the work reported in this paper.

## Appendix A Supplementary data

Supplementary figure 3

Supplementary table 1 Distribution of gastric cancer samples in each molecular subtype and Lauren classification, both counted according to two or four categories, which is related to Figure 1 C-F.

Supplementary table 2 Statistical tests between diffuse and non-diffuse types and GS and non-GS subtypes.

Supplementary table 3 Enrichment analyses of genes showing significant differences in GS to non-GS, and those in diffuse to non-diffuse types, using the Gene Ontology, KEGG, Reactome and WikiPathway databases.

Supplementary table 4 Enrichment analyses of most significant genes in GS, non-GS, diffuse and non-diffuse types, using the Gene Ontology, KEGG, Reactome and WikiPathway databases.
