# Supplementary figures and images for: Genetic profiling for diffuse type and genomically stable subtypes in gastric cancer
Source: Comput Struct Biotechnol J. 2020 Oct 29;18:3301–8. doi: 10.1016/j.csbj.2020.10.021 (PMC7666323; doi:10.1016/j.csbj.2020.10.021)

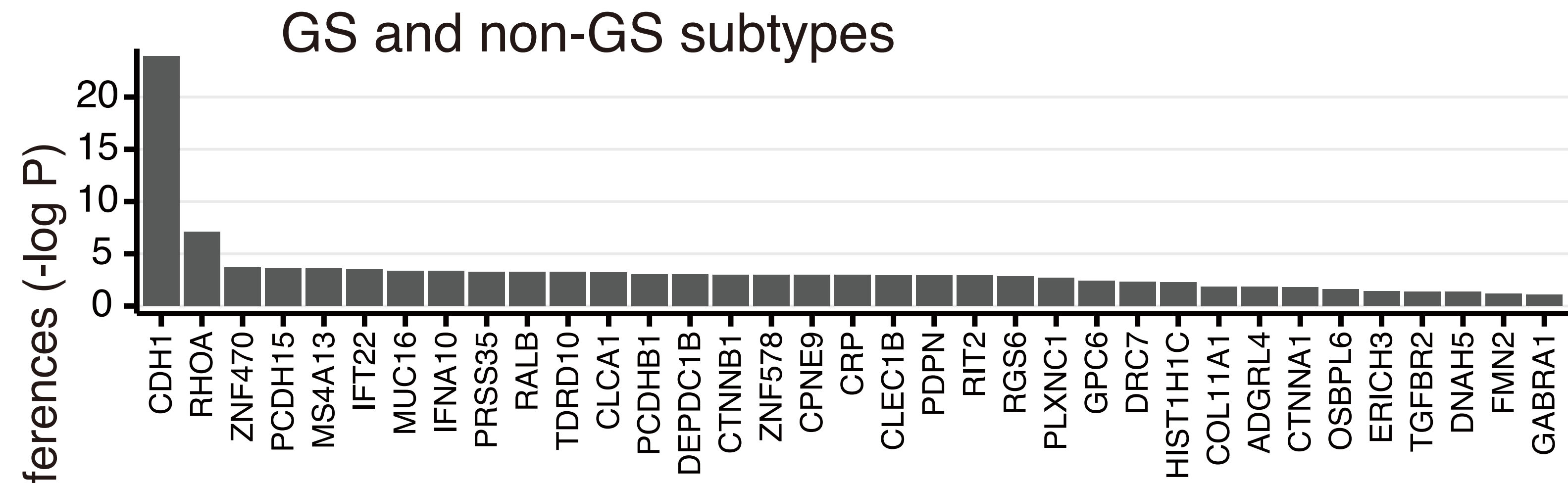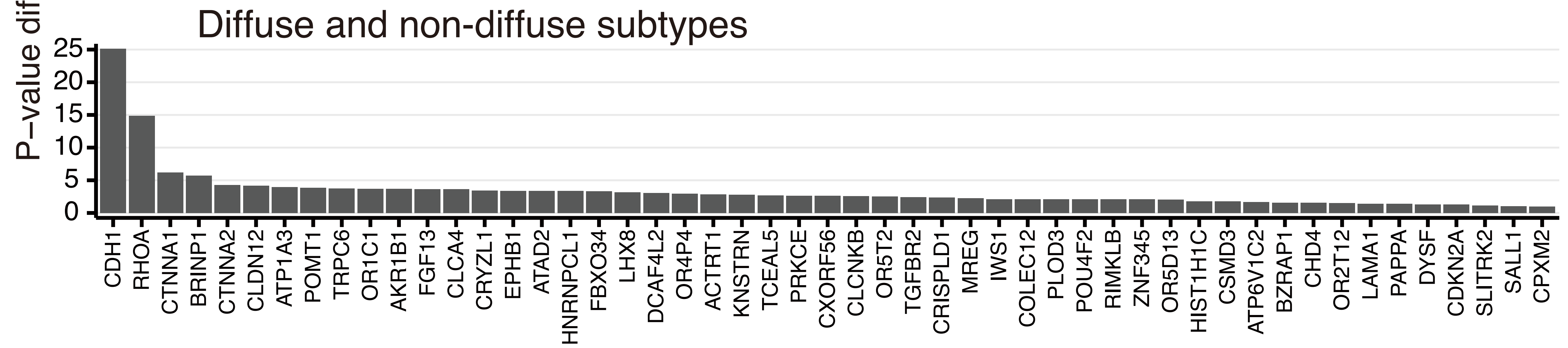

Supplement: Supplementary figure 1 [file mmc1.pdf]

Significantly mutated genes

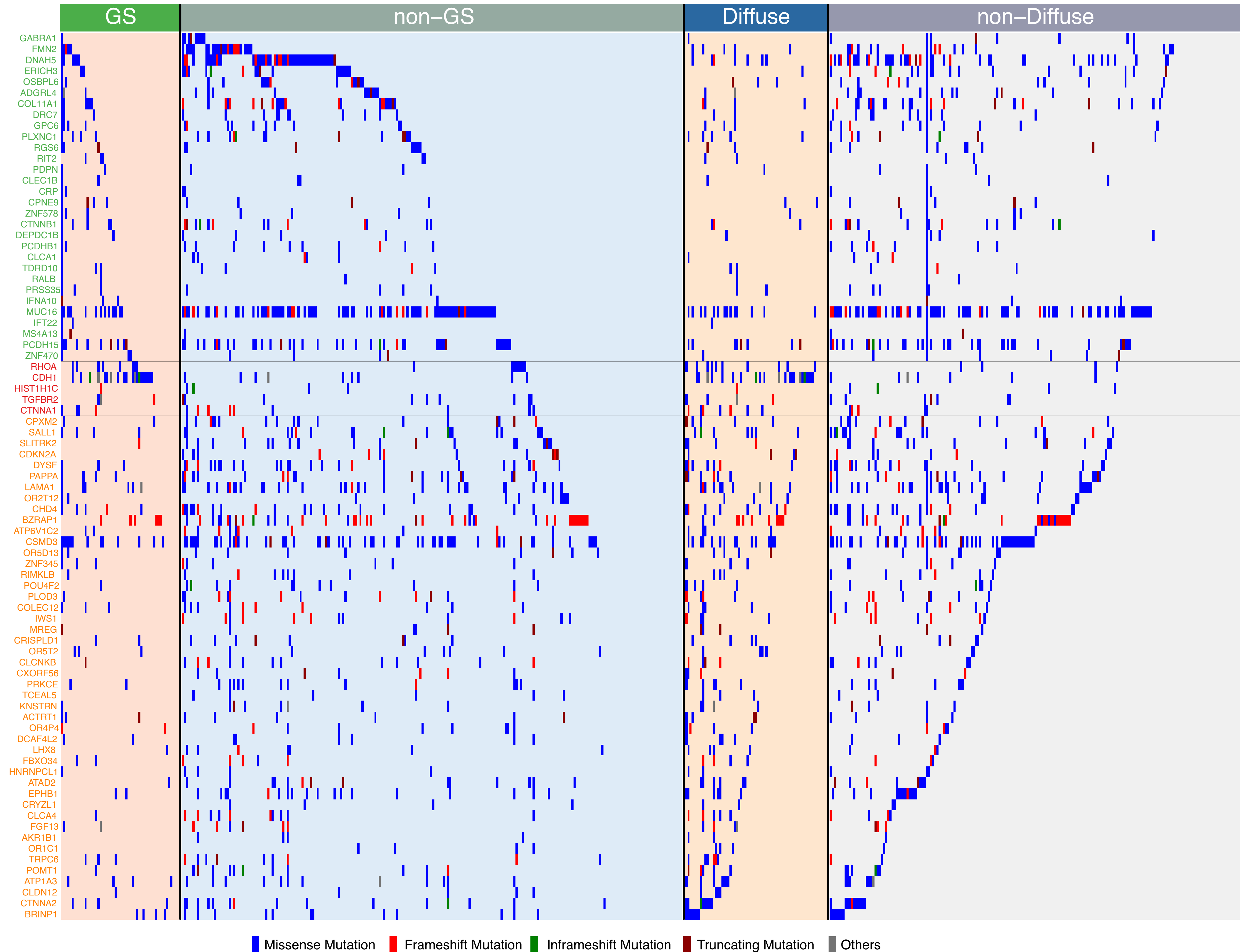

Supplement: Supplementary figure 2 [file mmc2.pdf]

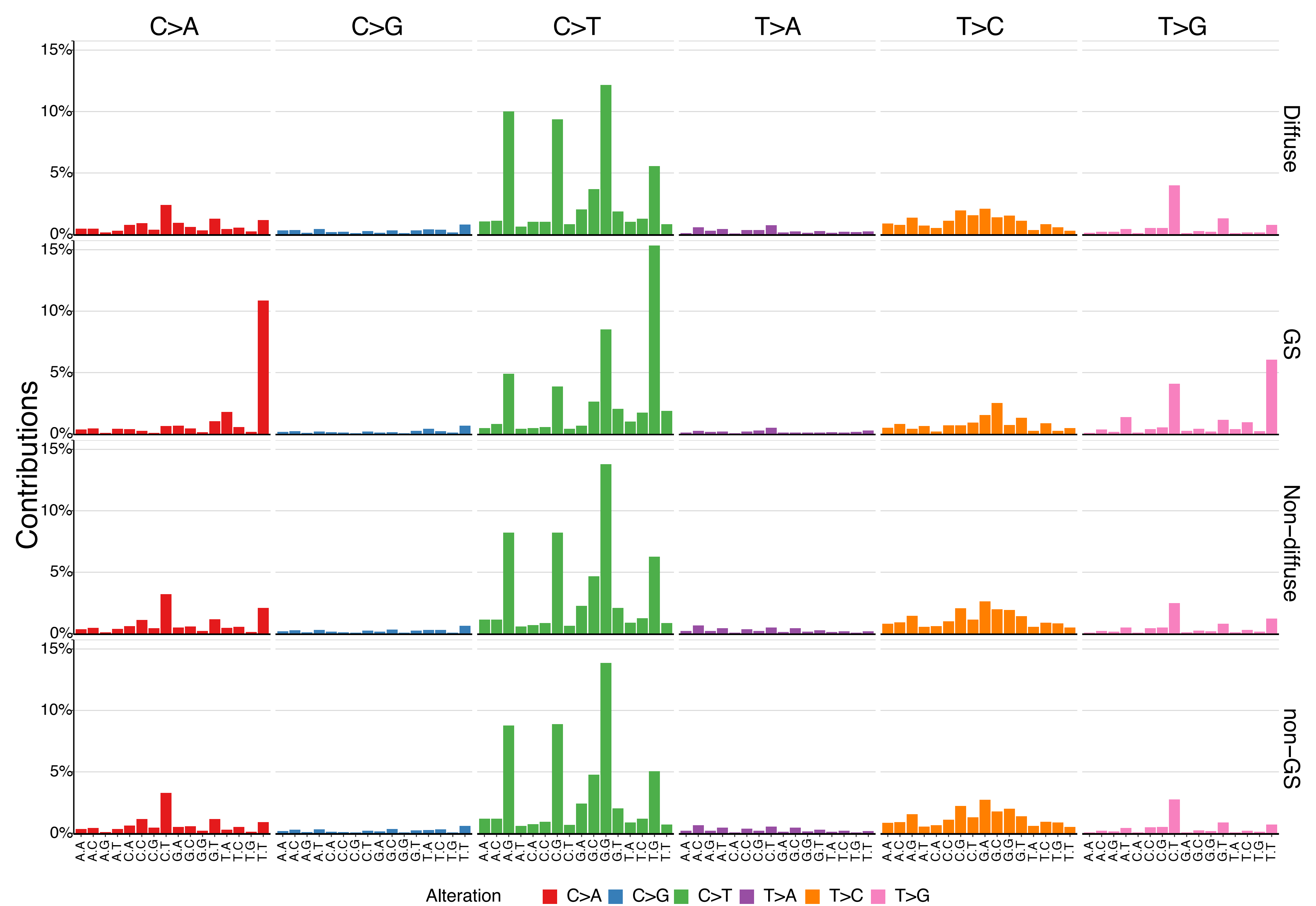

Supplement: Supplementary figure 3 [file mmc3.pdf]
